# Editorial: Intersection of adolescent sexual, reproductive, and mental health in Sub-Saharan Africa

**DOI:** 10.3389/frph.2025.1614317

**Published:** 2025-05-29

**Authors:** Negussie Boti Sidamo, Sultan Hussen Hebo, Stephen O. Chukwudeh, Zacharie Tsala Dimbuene

**Affiliations:** ^1^ School of Public Health, College of Medicine and Health Sciences, Arba Minch University, Arba Minch, Ethiopia; ^2^ Faculty of Social Sciences, Federal University, Oye-Ekiti, Nigeria; ^3^ School of Population and Development Sciences, University of Kinshasa, Kinshasa, Democratic Republic of Congo

**Keywords:** editorial, adolescent health, sexual and reproductive health, mental health, integrated services, Sub-Saharan Africa

**Editorial on the Research Topic**
Intersection of adolescent sexual, reproductive, and mental health in Sub-Saharan Africa

## Introduction

Adolescence is a critical developmental stage marked by significant physical, emotional, and social changes (1). In sub-Saharan Africa (SSA), over 60% of the population is under the age of 25, underscoring the urgent need to invest in adolescent health—particularly sexual, reproductive, and mental health (SRMH)—as both a public health imperative and a strategic pathway to sustainable development (2, 3). These domains are deeply interconnected: untreated mental health challenges can increase vulnerability to risky sexual behaviours, while adverse sexual and reproductive experiences—such as sexual violence, unplanned pregnancies, and restricted autonomy—can negatively affect mental well-being, with long-lasting consequences into adulthood (4, 5).

Despite their significance, adolescent SRMH issues continue to be underprioritized, underfunded, and poorly integrated into health systems across the region (6, 7). Structural, cultural, and policy-related barriers further limit access to youth-friendly services and comprehensive SRMH education (8). This Research Topic seeks to address these critical gaps by bringing together a collection of studies that explore the multifaceted and often overlooked intersection of SRMH among adolescents in SSA. The articles present diverse methodological approaches and rich contextual insights that collectively enhance our understanding of adolescent SRMH and provide evidence to inform future directions in research, policy, and practice.

## Overview of contributions

This Research Topic brings together eleven peer-reviewed articles from Ethiopia, Rwanda, the Democratic Republic of Congo (DRC), and Kenya, employing a variety of methodologies including quantitative, qualitative, mixed-methods designs, systematic reviews, and scoping reviews. Collectively, these studies provide a comprehensive understanding of adolescent and youth SRH in sub-Saharan Africa, revealing persistent challenges and highlighting evidence-based policy and programmatic recommendations.

Several articles examine sexual violence and its associated psychosocial consequences. A case-control study from Southern Ethiopia revealed a high prevalence of childhood sexual abuse, significantly associated with early exposure to violence, substance use, and lack of parental supervision. These findings emphasize the need for integrated child protection systems and early intervention programs aimed at mitigating adverse childhood experiences and fostering safe, supportive home environments (Dinagde et al.). Similarly, a study from the Democratic Republic of Congo (DRC) found a high incidence of non-consensual sexual acts among adolescents, reinforcing the urgency of enacting and enforcing robust legal frameworks and community-based mechanisms to prevent sexual violence and provide support to survivors (Yode et al.).

The social determinants of adolescent SRH are also a critical focus. In Rwanda, analysis of national survey data identified low levels of education, early sexual initiation, and rural residence as key drivers of teenage pregnancy. These findings underscore the importance of expanding comprehensive sexuality education (CSE), particularly in rural schools, and increasing girls' access to secondary and higher education to delay early childbearing and empower adolescent girls (Nduhuye et al.).

Risky sexual behavior among specific population groups was also explored. A study among taxi drivers in northwest Ethiopia revealed patterns influenced by alcohol consumption, peer pressure, and limited access to SRH information. These insights suggest the need for targeted behavior change communication strategies and the deployment of mobile SRH services tailored to high-risk occupational groups (Laikemariam and Fetene).

In the higher education context, a study among university students in eastern Ethiopia found alarmingly low levels of knowledge regarding reproductive rights, particularly among first-year students. This calls for the integration of reproductive rights education into university orientation and life skills training programs to enhance informed decision-making among young adults (Hussen et al.).

Service utilization barriers were highlighted in a systematic review and meta-analysis of high school students across Ethiopia, which reported low use of SRH services due to negative provider attitudes and the lack of youth-friendly services. This underscores the need for the Ministry of Health and educational institutions to expand access to adolescent- and youth-friendly health services that are confidential, accessible, and non-judgmental (Delie et al.).

A cross-sectional study in Gondar further illustrated the limited nature of parent–adolescent communication on SRH matters. Parental education and prior exposure to SRH education were identified as positive predictors of such communication. These findings point to the importance of national strategies that promote family life education and facilitate community-level dialogue to foster intergenerational communication on SRH (Melese et al.).

Qualitative research from Ethiopia revealed that adolescent sexual behaviors are significantly influenced by peer pressure, romantic relationships, and weak adult guidance. This highlights the need for school- and community-based mentorship programs to support adolescents in building life skills and making informed decisions (Baraki and Thupayagale-tshweneagae). Additionally, a multilevel and latent class analysis in the Gamo Zone revealed disparities in SRH service utilization based on residential location and family involvement, indicating the importance of equity-focused health system planning and family-inclusive adolescent health promotion (Sidamo et al.).

At the regional level, a scoping review of SRH interventions in sub-Saharan Africa found that while youth-friendly services, peer education, and school-based programs have demonstrated impact, implementation gaps remain significant. This highlights the need for stronger monitoring and evaluation frameworks and sustained investment in capacity-building for SRH program implementers (Chipako et al.). Finally, a study from Kenya emphasized adolescents' preferences for contraceptive services that are private, respectful, and convenient. These insights advocate for adolescent-centered service delivery models that are confidential, responsive to user preferences, and designed to meet the unique needs of young people (Harrington et al.).

Together, these contributions provide an in-depth, evidence-based understanding of the diverse SRH challenges faced by adolescents and youth across sub-Saharan Africa. They call for multisectoral, rights-based, and context-specific approaches that prioritize equity, inclusivity, and the meaningful engagement of young people. To achieve sustainable progress in adolescent and youth SRH outcomes, policy and program efforts must be coordinated, responsive, and firmly grounded in the lived experiences of the region's adolescents.

## Emerging themes and synthesis

Collectively, these studies surface several cross-cutting themes with implications for adolescent health programming and policy in SSA:

1. Integrated SRMH Services: The interconnected nature of sexual, reproductive, and mental health calls for integrated, adolescent-centered service delivery models. The psychosocial aftermath of sexual violence and unplanned pregnancies, compounded by limited access to care, demands holistic approaches that address both physical and mental well-being.
2. Gender Disparities: Adolescent girls continue to bear the brunt of adverse SRMH outcomes, including coerced sex, early pregnancies, and societal stigma. Effective interventions must adopt a gender-responsive lens that promotes equity, empowerment, and protection.
3. Socio-cultural and Structural Barriers: Taboos around sexuality, restrictive gender norms, limited parent–child communication, and structural inadequacies within health systems undermine adolescent access to essential services and information. Culturally sensitive and community-based interventions are essential to overcome these challenges.
4. Adolescent Participation and Contextualization: Meaningful engagement of adolescents in the design, implementation, and evaluation of programs is crucial. Interventions must be context-specific and grounded in the lived realities of diverse adolescent populations.

## Critical gaps and future directions

While this Research Topic contributes valuable evidence, several gaps still persist:

• Marginalized Adolescents: There remains a paucity of research focusing on adolescents from marginalized backgrounds, including those with disabilities, those living in humanitarian settings, and sexual and gender minorities. Targeted research is needed to understand and respond to their specific vulnerabilities.
• Mental Health Integration: Mental health remains understudied within SRH frameworks. Evidence underscores that mental health is both a determinant and consequence of SRH outcomes and it must be systematically integrated into adolescent health policies and programs.
• Research-to-Policy Translation: A recurring challenge is the disconnect between research findings and their translation into policy and scalable interventions. Strengthening implementation science, fostering multi-sectoral collaboration, and ensuring adequate funding are essential to bridge this gap.

Future research should prioritize longitudinal studies to track SRMH outcomes over time, robust intervention trials to assess efficacy in real-world settings, and implementation research to support the uptake of evidence-based practices.

## Conclusion

This collection of SRMH-related papers represents a significant step forward in illuminating the multidimensional and interrelated nature of adolescent SRMH in SSA. We extend our sincere appreciation to the contributing authors for their scholarly rigor, to the reviewers for their constructive feedback, and to all those who supported the development of this Research Topic.

As SSA continues to grapple with the dual challenges of a youthful population and constrained health systems, it is essential to prioritize integrated, inclusive, and evidence-based adolescent health strategies. The future of the continent depends on the well-being of its adolescents—not only in terms of their reproductive health, but also their mental wellness, identity, and aspirations. The health and dignity of young people in SSA are not optional—they are fundamental human rights and critical pillars for sustainable development.
